# Featuring long-loop tournaments: breeding and training profiles and blood changes in criollo horses before and after exercise

**DOI:** 10.29374/2527-2179.bjvm005723

**Published:** 2024-10-11

**Authors:** Andrielli Trentim Pereira, Ricardo Pozzobon, Bruno Leite dos Anjos, Alfredo Rafael Kunz, Leonardo Trentin Chaves, Erika Carla Smilgys, Bárbara da Silva Andrade, Vinícius Leobet Lunkes, Cinthia Melazzo de Andrade, Márcio Machado Costa

**Affiliations:** 1 Veterinarian, Programa de Pós-Graduação em Ciência Animal, Departamento de Clínica de Grandes Animais (DCGA), Hospital Veterinário Universitário (HUVet), Universidade Federal do Pampa (UNIPAMPA), Uruguaiana, RS, Brazil; 2 Veterinarian, DSc. DCGA, Hospital Universitário Veterinário (HVU), Universidade Federal de Santa Maria (UFSM), Santa Maria, RS, Brazil; 3 Veterinarian, DSc. Departamento de Patologia Veterinária (DPV), HUVet, UNIPAMPA, Uruguaiana, RS, Brazil; 4 Undergraduate in Veterinary Medicine, HUVet, UNIPAMPA, Uruguaiana, RS, Brazil; 5 Undergraduate in Veterinary Medicine, HVU, UFSM, Santa Maria, RS, Brazil; 6 Veterinarian, MSc. Departamento de Patologia Clínica Veterinária (DPCV), HVU, UFSM, Santa Maria, RS, Brazil; 7 Veterinarian, DSc. DPCV, HVU, UFSM, Santa Maria, RS, Brazil; 8 Veterinarian, DSc. DPCV, Universidade Federal de Uberlândia (UFU), Hospital Veterinário (HV), Uberlândia, MG, Brazil

**Keywords:** biochemical changes, equine, exercise, hematological changes, alterações bioquímicas, equino, exercício, alterações hematológicas

## Abstract

Long-loop rodeo is a major competition for Criollo horses. We aimed to feature long-loop tournaments and to assess the profiles of competing horses. The animals (n = 49) were registered, and their body mass/scores, information about breeding, feeding, and training management, loaded weight, and tournament track (n = 11) were collected; speeds reached were estimated. Heart and respiratory rates of horses (n = 27) were collected before the tournament, on the final day of the event, and 18-24 h after the end of the tournament. Blood samples were collected from the animals at the same time. Blood count (n = 19) and biochemical profile (n = 28) were conducted based on total proteins, albumin, globulin, phosphorus, sodium, potassium, urea, creatinine, glucose, triglycerides, and cholesterol. Data were assessed using analysis of variance in association with Tukey’s test (P ≤0.05) and Spearman's correlation (P ≤0.05). Most horses were bred in a semi-stable system, fed roughage and concentrate, and subjected to non-standardized training. The tournament comprised a high-intensity and short-duration exercise with a mean speed of 6.44 m/s, during which the horses carried 25.59% of their body weight on their backs. Clinical evaluations and hematological and biochemical assessments in this study showed physiological changes caused by exercise.

## Resumo

O rodeio de laço comprido é uma das principais competições em que o cavalo Crioulo participa. O objetivo deste trabalho foi caracterizar a competição e o perfil dos equinos competidores. Foram cadastrados os animais (n=49) com a mensuração de massa e escore corporal e coletadas as informações sobre criação, alimentação e treinamento, peso do cavaleiro e da sela, dados da pista (n=11) e velocidade estimada dos animais. Nos tempos: 24 horas pré-prova, após a última corrida do último dia de prova e entre 18 a 24 horas após o final da prova, foram aferidas as frequências cardíaca e respiratória dos equinos (n=27) e a temperatura ambiente, foram coletadas amostras sanguíneas dos animais nos mesmos tempos para realização de hemograma (n=19) e bioquímicos (n=28) abrangendo proteínas totais, albumina, globulina, fósforo, sódio, potássio, ureia, creatinina, glicose, triglicerídeos e colesterol. Os dados foram avaliados por análise de variância associada ao teste de Tukey (P ≤0,05) e submetidos à correlação de Spearman (P ≤0,05). Os equinos eram, majoritariamente, criados em sistema de semi-estabulação, alimentados com volumoso e concentrado e com treinamento não padronizado. A prova mostrou-se um exercício de alta intensidade e curta duração, em pistas de areia, com velocidade média de 6,44 m/s e os equinos carregaram 25,59% do seu peso corporal. As avaliações clínicas, hematológicas e bioquímicas demonstraram alterações fisiológicas causadas pelo exercício, indicando aptidão.

## Introduction

The first Criollo horses settled in Argentina, Chile, Uruguay, Paraguay, Peru, and Southern Brazil, where they lived freely (Associação Brasileira de Criadores de Cavalos Crioulos, 2018). They encountered challenges, such as adverse feeding and temperature conditions, which resulted in remarkable rusticity and resistance (Vidart, 2004).

Long-loop competition, which has great cultural and economic relevance, is a modality that most often practiced with Criollo horses. It simulates the work performed in the field during roping and immobilizing bovine cattle for treatment, at a mean distance of 100 m, in enclosed areas (Associação Brasileira de Criadores de Cavalos Crioulos, 2014).

The athletic profile of the physiological and biochemical changes in Chilean Criollo horses participating in Chilean rodeo events has already been described (Pérez et al., 1997). However, although most Criollo horses in Brazil participate in long-loop tournaments, there is limited data on their athletic profile. Furthermore, it is unclear whether exercise can lead to reduced athletic performance and affect horses’ well-being.

The evolution of Criollo horses as high-performance athletes and the notable rise in this breed are study subjects. Moreover, determining the hematological and biochemical biomarkers of equine athletes can enhance understanding of the impacts of nutrition and exercise regimens and aid in detecting changes in their health status (Vazzana et al., 2014).

Therefore, in this study, we aimed to feature long-loop tournament data, describe the breeding and training profiles of competing Criollo horses, and assess the impact of physical activity on their hematological and biochemical profiles.

## Materials and methods

The inclusion criteria were male and female animals of different age groups, who were registered in the genealogical record of the ABCCC and who participated in a 2 d long-loop tournament.

The inclusion procedures for animal and rider registration comprised an interview and animal inspection to gather information such as age, body mass, body condition score, and breeding and feeding management. Overall 49 animals and riders were evaluated, excluding the body condition score, which was applied to 33 horses. The profile of competing horses and long-loop tournament were featured based on a larger number of animals than those used for blood collection and for heart rate (HR) and respiratory rate (RR) assessments to enrich the study.

Animals’ mass was estimated with the aid of a weighing tape; their body condition score was determined through a scale from one (very thin) to nine (very obese), according to Henneke et al. (1983).

Long-loop competitions are 1-3 d or longer. The purpose of the tournament was to tie a bovine at a distance of ≤100 m by involving only its horns and ears for a valid loop. Whether the loop does not reach such a region or it involves the entire head and neck of the animal, the run is considered lost, and competitors do not score points. The horse and rider remained on standby, waiting for the bovine to run from the beginning of the track to be tied before crossing 100 m. After the loop is performed, they reach the end of the track and return to the starting point for another run; however, this process occurs in varying time intervals because it depends on the number of competitors who remain in the run.

**Figure 1:**
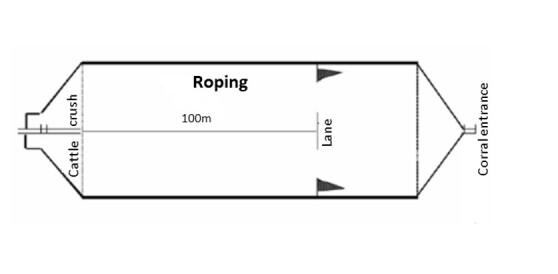
Schematic drawing of the tournament track. The beginning (cattle crush), the maximum limit to tie the bovine (lane) and the end (corral entrance).

The long-loop track (Figure 1) starts at “cattle crush,” the maximum limit for the bovine to be roped is called “lane” and end of the track is the point where the bovine is restrained for loop removal and to be driven to the corral.

Eleven tournament tracks were evaluated; their lengths were measured using a tape measure, and their depths were measured with a ruler at three different points along the tracks for mean calculation.

The tournament was concentrated on 2 d (Saturday and Sunday), with the finals on the second day. The number of runs in which the 41 horses participated was recorded during the two competition days. Each run was timed to determine the horses’ mean speed during the competition. The timing started when the horses entered the track along with the bovine to be tied until the end of the run, which corresponded to the time when the bovine entered the corral.

Animals (n= 27) had their RR and (HR assessed 24 hours before the tournament, that is at rest (T0), immediately after the last run of the final tournament day (T1), and 18-24 h after the end of the tournament (T18).

Ten milliliters of blood was collected from the jugular vein of each horse participating in the tournament and subjected to hematological (19 animals, because some samples did not meet the minimum quality control requirements) and biochemical (28 animals) analyses. Samples were collected placed in tubes filled with ethylenediaminetetraacetic acid - EDTA (for complete blood count), sodium fluoride (for glucose analysis), and without anticoagulant (for the remaining biochemical analyses). They were collected simultaneously during HR and RR assessments. The 6 h time interval at T18 is due to logistical challenges: horses finishing the second tournament day at similar times were housed in distant locations, complicating the precise sample collection at designated times.

A veterinary hematology analyzer (BC 2.8 MYND Hematoclin) was used to determine the erythrocyte and leukocyte counts and hemoglobin concentration. Hematocrit was manually determined, and fibrinogen was determined using the heat precipitation method and subsequent refractometer readings. Blood smears were prepared and stained with A veterinary hematology analyzer (BC 2.8 MYND Hematoclin) was used to determine the erythrocyte and leukocyte counts and hemoglobin concentration. Hematocrit was manually determined, and fibrinogen was determined using the heat precipitation method and subsequent refractometer readings. Blood smears were prepared and stained with diff-quick stain to enable differential leukocyte counts and morphological observations of blood cells.

Total proteins (TP), albumin, electrolytes such as phosphorus (P), sodium (Na^+^) and potassium (K^+^), urea, creatinine, glucose, triglycerides, and cholesterol were assessed using commercial kits, such as Quibasa-Bioclin®, and automated equipment (BS-120, MINDRAY). Globulin levels were calculated by subtracting the albumin value from the TP level.

Statistical analysis was performed using the SPSS12 statistical package Prism (GraphPad Prism, Version 5®).

Results were expressed as means and analysis of variance was performed, in association with Tukey’s multiple comparison test, at a significance level of P ≤0.05.

In addition, Spearman’s correlation was performed at a significance level of P ≤ 0.05 to assess the intensity of correlation between some variables. Collection time deltas were calculated—for example, mean T1 values minus mean T0 values and mean T18 values minus mean T0 values—to reduce the distribution and approximate variables. Data, such as the number of runs and training, were categorized from 1 to 4 and 0 to 3, respectively. The results were presented as scatter plots.

## Results

The mean animal age was 7.2 ± 2.7 years; 36.7% (18/49) of males were neutered, whereas females comprised 63.2% (31/49) of the evaluated animals. The mean mass of animals reached 422.2 ± 35.8 kg. The body condition score results were distributed as follows: 3.0% of the animals scored 5 (1/33), 21.2% scored 6 (7/33), 60.7% scored 7 (20/33), and 15.1% scored 8 (5/33).

Most animals investigated in the current study (79.6% [39/49]) were bred under a semi-stable system, 20.4% (10/49) were free in the native field, 60% (06/10) were fed on native pasture, and their diet was supplemented with extra roughage (*Medicago sativa* hay) and concentrate. The roughage in the diet comprised native fields, *Medicago sativa* hay, *Avena sativa*, or *Lolium multiflorum* pasture, depending on the availability of the farm or breeder. The diet concentrate comprised commercial feed containing cereals, amino acids, vitamins, and minerals, 3,200 kcal/kg of digestible energy, and *A. sativa*, and *Zea mays* in the grains. The amount of concentrate supplied to the animals ranged 3-7 kg, on average, that is, from 0.7% to 1.6% of the live weight per animal, two to three daily. With respect to mineral supplementation, 30.6% (15/49) of the animals received common and mineralized salt *ad libitum*.

Training management evaluation showed that 69.4% (34/49) of the animals were trained once or twice a week, 16.3% (08/49) trained three to four times a week, 10.2% (05/49) trained more than four times a week, and 4.0% (02/49) did not train at all. Despite the lack of a standardized frequency, training sections comprised 10-15 runs (each) on similar tracks or at the same places where the tournament was held.

The floors of all 11 evaluated tracks were composed of sand, with a mean depth of 7.4 ± 1.2 cm. The mean length of the cattle crush up to the corral entrance was 155 ± 38.2 m (Figure 1).

The investigated animals performed 12.7 runs (on average) in the two rodeo days; it covered a total distance of 3,658 ± 2,267 m, on average. The mean speed of the evaluated horses was 6.4 ± 0.6 m/s.

The mean mass carried by the evaluated horses was 90.8 ± 14.6 kg (for riders) and 16.8 ± 2.8 kg (for saddles), which totaled 107.6 kg carried on their back; this weight corresponded to 25.6 ± 3.9% of the mean body mass of the animals.

**Figure 2:**
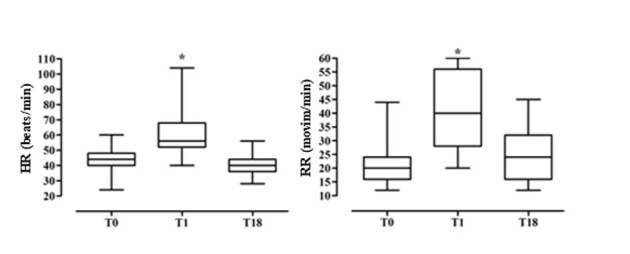
Mean, maximum and minimum HR and RR values recorded 24h before the tournament (T0), right after the last run of the last tournament (T1) and 18 to 24h after of the end of the tournament (T18) for Criollo horses participating in two-day long-loop rodeo. *Significant differences, at P ≤ 0.05.

The HR and RR evaluated were normal at T0; however, they significantly increased at T1 and returned to baseline values at T18 (Figure 2).

Tables 1 and 2 list the hematological and biochemical data of the evaluated animals. A significant increase was noted in the number of erythrocytes, hemoglobin, and hematocrit immediately after the last run in T1; a similar outcome was observed for TP. A significant increase was noted in creatinine and cholesterol levels in T1 and T18.

**Table 1:** Mean values, standard deviation (SD) and maximum and minimum erythrogram, fibrinogen and leukogram values recorded in T0, T1 and T18 for Criollo horses participating in two-day long-loop tournaments.

	**T0**	**T1**	**T18**
**Hematocrit (32-52%)**			
Mean	33.36^b^	38.64^a^	33.83^b^
SD	4.35	4.26	3.64
Min. and max. values	25-45.1	33-48.1	28.5-43.9
**Hemoglobin (11-19 g/dL)**	**T0**	**T1**	**T18**
Mean	11.53^b^	13.61^a^	11.44^b^
SD	1.21	1.31	1.31
Min. and max. values	9.4-15	11.3-16.2	9.8-15
**Erythrocytes (6.5-12.5x10^6^/µL)**	**T0**	**T1**	**T18**
Mean	7.42^b^	8.64^a^	7.38^b^
SD	0.77	0.71	0.78
Min. and max. values	6.23-8.84	7.61-9.88	6.32-8.82
**Fibrinogen (100-400 mg/dL)**	**T0**	**T1**	**T18**
Mean	242.1^a^	210.5^a^	236.8^a^
SD	150.2	172.9	167.4
Min. and max. values	0-600	0-600	0-600
**Total leukocytes (5.500-12.500 /µL)**	**T0**	**T1**	**T18**
Mean	9142^a^	14705^a^	13826^a^
SD	2088	19539	20461
Min. and max. values	6100-13300	7100-13100	6300-13400
**Neutrophils (2.700-6.700 /µL)**	**T0**	**T1**	**T18**
Mean	4895^a^	8885^a^	5227^a^
SD	1207	11541	1488
Min. and max. values	3223-8512	3237-10140	3213-8424
**Lymphocytes (1.500-5.500 /µL)**	**T0**	**T1**	**T18**
Mean	3848^a^	3544^a^	3442^a^
SD	1704	1451	1461
Min. and max. values	2232-7812	1349-7869	1764-8487

Different letters on the same line indicate significant differences, at P≤0.05.

**Table 2:** Mean values, standard deviation (SD) and maximum and minimum TP, albumin, globulin, P, Na^+^, K^+^, creatinine, urea, glucose, cholesterol and triglycerides levels recorded in T0, T1 and T18 for Criollo horses participating in two-day long-loop tournaments.

	**T0**	**T1**	**T18**
**Total Protein (5.2-7.9 g/dL)**			
Mean	6.7^b^	7.03^a^	6.83^ab^
SD	079	0.63	0.60
Min. and max. values	4.8-8.2	5.9-9	5.6-8.1
**Albumin (2.4-4.1 g/dL)**	**T0**	**T1**	**T18**
Mean	3.04^a^	3.19^a^	3.2^a^
SD	0.44	0.41	0.71
Min. and max. values	2.3-3.93	2.5-3.96	2.6-6.1
**Globulin (2.6-4.6 g/dL)**	**T0**	**T1**	**T18**
Mean	3.76^ab^	3.97^a^	3.52^b^
SD	0.66	0.57	0.85
Min. and max. values	2.4-4.87	3.1-5.44	0.3-4.9
**Phosphorus (1.5-4.7 mg/dL)**	**T0**	**T1**	**T18**
Mean	3.46^a^	3.85^a^	3.77^a^
SD	0.69	0.65	0.63
Min. and max. values	2.5-5.3	2.6-4.9	2.7-4.9
**Sodium (132-146 mmol/L)**	**T0**	**T1**	**T18**
Mean	143.4^a^	140.6^a^	142.5^a^
SD	25.35	7.80	3.98
Min. and max. values	93.1-238.6	110.3-148.4	132.7-150.9
**Potassium (2.4-4.6 mmol/L)**	**T0**	**T1**	**T18**
Mean	3.69^a^	3.58^a^	3.31^a^
SD	2.37	0.37	0.31
Min. and max. values	1.7-13.6	2.9-4.1	2.6-3.9
**Creatinine (1.2-1.9 mg/dL)**	**T0**	**T1**	**T18**
Mean	1.49^a^	1.71^b^	1.61^c^
SD	0.23	0.17	0.18
Min. and max. values	1-1.9	1.4-2.09	1.3-2.01
**Urea (21-51 mg/dL)**	**T0**	**T1**	**T18**
Mean	42.18^a^	43.25^a^	44.36^a^
SD	10.23	7.91	9.25
Min. and max. values	25-74	29-59	29-72
**Glucose (75-115 mg/dL)**	**T0**	**T1**	**T18**
Mean	100.4^a^	97.79^a^	92.18^a^
SD	22.50	18.38	9.44
Min. and max. values	79-185	71-179	76-113
**Cholesterol (75-150 mg/dL)**	**T0**	**T1**	**T18**
Mean	92.11^b^	97.64^a^	96.11^ab^
SD	17.60	17.28	18.17
Min. and max. values	62-125	67-132	64-136
**Triglycerides (4-44 mg/dL)**			
Mean	27.14^a^	29.96^a^	37.11^a^
SD	13.26	10.80	30.85
Min. and max. values	9-73	19-63	9-147

Different letters on the same line indicate significant differences, at P≤0.05.

## Discussion

We believe that the data analyzed herein are the first set of information on the long-loop rodeo and profile of Criollo horses participating in this modality.

The body condition scores of the vast majority of the evaluated animals (32/33) were above the ideal value for other breeds of equine athletes (Rooney, 2000). However, it met the body standards indicated by the ABCCC, since Criollo horses present a good muscular lining, strong and muscular croup, full flanks, a well-inserted tail, and always covered ribs (Associação Brasileira de Criadores de Cavalos Crioulos, 2018), which enables higher body scores than those of other breeds. Body condition score is a subjective body status indicator applied to horses. It is based on fat deposits observed in certain regions through visual assessment of the animal and palpation (Gobesso et al., 2014). The ideal score established for horses, considering the scale from one to nine (Henneke et al., 1983), ranges from 4 to 5, according to which ribs may be difficult to see but easy to palpate (Rooney, 2000). Criollo horses bred under intensive systems easily accumulate fat deposits, leading to undesirable consequences, such as metabolic syndrome (Cantarelli et al., 2018) and a higher likelihood of presenting with chronic degenerative joint injuries capable of compromising their athletic future (Amaral et al., 2017).

The adopted training management (69.4% of animals trained once or twice a week) appears to be effective for types of exercise practiced. It is crucial to emphasize the main goal as training riders to handle the rope loop and improve their physical fitness and ability to handle bovines. As the long loop is not exclusively a professional sport, there is lack of standardized training. Regarding animals that did not train at all (4.0%), we speculate that some form of adaptation may have occurred considering their longstanding involvement in this sports modality. They were selected for their ability to perform strength- and speed-demanding activities, such as fieldwork carried out with cattle (Pérez et al., 1997).

In the semi-stall system which covered the majority (79.6%), animals only spend the night in stables, which is why roughage intake is not measured by owners and keepers, and roughage feeding was supplemented with *M. sativa* hay. Some animals may have received larger amounts of grain than recommended. The forage supply to horses should comprise 1 kg of dry matter every 100 kg of body weight per day; the remainder of its requirements should be provided by concentrate, which should not exceed 50% of the diet (Lewis, 2000).

The frequency of grain supply per day is within the ideal range as the ideal frequency of the supplied grains is two to three times a day (Lewis, 2000). The breeding system for these animals appeared to be as natural as possible.

Based on the digestible energy values of the commercial feed provided to the evaluated animals, they were fed slightly more than necessary for daily maintenance. The remaining necessary energy was likely supplemented by diet (roughage and fresh grains). Providing digestible energy to horses involved in intense exercise doubles their maintenance value, corresponding to 15,000 kcal/day (Lewis, 2000; Rooney, 2000).

Few evaluated animals (30.6%) received mineralized salt; however, equine athletes lose electrolytes during sweat replacement, thus, this supplementation is of paramount importance (Lewis, 2000). Raising awareness among animal keepers and owners regarding the importance of providing this supplement to athletic animals should be emphasized. The dry matter in athlete horse feed must contain 0.3% sodium chloride; thus, 0.5-1% salt must be added to the grain mix provided to these animals to meet this requirement; it can also be provided *ad libitum* (Lewis, 2000).

The mean track depth did not reach 10 cm. Considering the sports modality investigated is semi-professional, there is limited attention to or standardization of track depth. Studies carried out on various surface types, such as mats, sand, and concrete tracks, have shown that the surface of the tracks often used in equestrian competitions affects animal performance associated with locomotion dynamics, such as hoof landing speed and joint flexion and extension (Burn & Usmar, 2004; Mendez-Angulo et al., 2014). Therefore, we emphasize that care must be taken to maintain the depth of long-loop tracks to avoid likely injuries in the distal joints of animal limbs, since 10-cm-deep sandy soil is better than hard soil for proper flexion of the tarsus and fetlock joints (Mendez-Angulo et al., 2014).

Mean speed results recorded for Brazilian Criollo horses (6.4 ± 0.6 m/s) were close to the mean speed recorded for Chilean Criollo horses participating in typical rodeos (6.9 ± 1.1 m/s) (Pérez et al., 1997). The mean speed of horses in long-loop competitions varies considerably because it directly depends on bovine speed. The mean speed recorded in the current study showed that the exercise in question requires a burst of energy at the beginning of each run and demands anaerobic energy, as it is a high-intensity and short-duration activity (Coelho et al., 2011).

The evaluated animals carried weight load (25.6 ± 3.9%) higher than that indicated in the literature for riding animals (Matsuura et al., 2013), which required an even greater physical effort and reinforced the hypothesis about these animals’ resistance when they practice intense exercises. A study in horses with a body score between 5 and 7, using the same rating scale as our research, concluded that animals carrying 23.6-27.5% of their body weight may experience temporary lameness and musculoskeletal pain (Dyson et al., 2020). This highlights the importance of raising awareness among animal owners and trainers about controlling the load carried by these animals. The National Racing Code in Brazil does not set a value for riders’ weight; however, the heaviest weight carried by racehorses cannot exceed 65 kg (minimum weight is 48 kg). Thus, the maximum weight allowed should be 13% of the body weight of a racehorse when considering a 500-kg horse. A study conducted with Japanese native horses measured, using an accelerometer, changes in asymmetry and regularity of horses carrying 80-130 kg of their total weight during step and trot. Results have shown significant differences when the animals carried 25-30% of their body weight (Matsuura et al., 2013).

**Figure 3:**
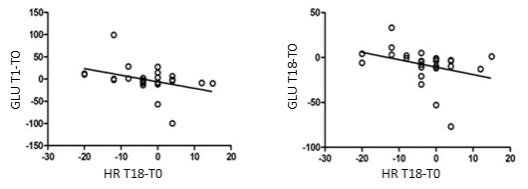
Significant moderate and negative correlations between glucose deltas (T1-T0 and T18-T0) and HR delta (T18-T0). Glucose values decreased as HR values increased in Criollo horses participating in two-day long-loop tournaments.

The increased HR and RR resulting from exercise were within the expected range, since changes resulting from exercise practice are normal (Gomes et al., 2019); their return to normal values at T18 showed that the evaluated animals presented good clinical recovery of cardiovascular and respiratory functions. Increased HR and RR after exercise can be attributed to sympathetic nervous system stimulation, leading to increased catecholamine (adrenaline) levels (Allaam et al., 2014). Increased metabolic, aerobic, and anaerobic glycolysis rates were also observed, leading to major changes in cardiovascular and respiratory functions. This outcome was demonstrated in the current study through significant moderate and negative correlations between HR (T18 - T0) and glucose (T1 - T0 and T18 - T0) deltas. Blood glucose levels decreased as HR values increased (Figure 3). Horses use their respiratory system to release heat generated during exercise (Allaam et al., 2014). This information substantiates the observed increases in HR and RR values.

**Figure 4:**
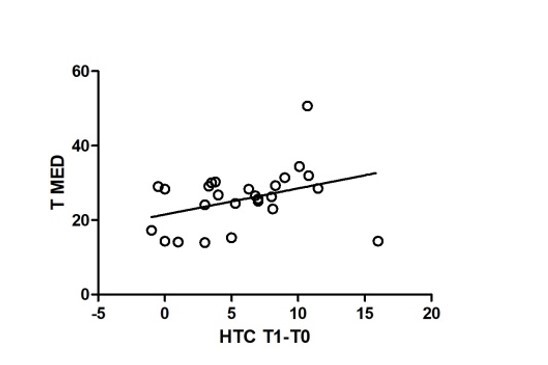
Significant moderate and positive correlation between the mean time of each run and hematocrit delta (T1-T0). As the mean time of each run increased, hematocrit values (T1-T0) also increased in Criollo horses participating in two-day long-loop tournaments.

Hemogram tests results of the 19 animals evaluated confirmed the good health status of the evaluated horses. This complementary exam aids in detecting abnormalities and allows for the evaluation of athletic performance. Thus, it serves as an important tool for adapting training programs and to enhance the physical performance of athletic horses (Assenza et al., 2015). The current study recorded a significant increase in erythrocyte, hemoglobin, and hematocrit rates at T1, although they remained within the reference values and returned to baseline values at T18. Similar results have been observed in jumping horses before and immediately after exercise (Dias et al., 2011). These changes are likely attributed to erythrocyte storage in the spleen during rest and release of catecholamines during exercise. This promotes splenic contraction and causes physiological erythrocytosis (Gomes et al., 2019; Stockham & Scott, 2011). This outcome was observed in the significant moderate positive correlation between hematocrit delta (T1 - T0) and animals’ mean time; horses presented longer mean time (that is, longer duration of each run) and recorded higher hematocrit values (Figure 4).

As the mean time of each run increased, hematocrit values (T1 - T0) also increased in Criollo horses participating in 2 d long-loop tournaments.

Based on the hemogram results, hemoconcentration due to splenic contraction stimulation appears to have occurred, as evidenced by T1. However, the increased erythrogram values did not persist until T18 or lead to significant changes in fibrinogen levels. The evaluated animals showed an increased TP at T1, suggesting fluid loss during exercise. TP concentrations in horses may indicate the incidence of post-exercise dehydration (Gondin et al., 2013). However, while TP values returned to their baseline levels at T18, neither TP nor hematocrit reached levels that would indicate dehydration, since a considerable increase in hematocrit can occur in dehydrated animals (Stockham & Scott, 2011). This indicates adaptation to the exercise type.

The leukogram results did not show significant differences, though they did show slight leukocytosis due to neutrophilia at T1. Leukogram values recorded in horseback riding animals at rest and after exercise showed significant differences in neutrophilia-associated leukocytosis and lymphocytosis (Dias et al., 2011). These occur in a physiological manner due to catecholamine release during exercise (Stockham & Scott, 2011). This finding suggests that the exercise practiced could not change leukocyte parameters, as it occurred during a horseback riding tournament, showing these animals’ adaptation.

As Na^+^, K^+^, and P ions remained unchanged and within the reference, the 28 did not experience electrolytic losses during exercise. Hydroelectrolytic issues are associated with abnormal fluid loss, as evidenced in Arab horses participating in endurance tournaments and in horses of different breeds participating in resistance tournaments (Dumont et al., 2012; Sow et al., 2016). Increased post-exercise P levels may be explained by extensive adenosine triphosphate destruction during exercise, resulting in physiological variation (Gomes et al., 2020). Electrolytes are lost through sweating during exercising, resulting in hyponatremia due to dilution caused by water intake after intense sweating, or hypokalemia due to fluid losses during sweating, since horses' sweat is rich in K^+^ (Moreira et al., 2015). Such losses suggest a lack of adaptation to exercise (Dumont et al., 2012), which was not observed in the animals evaluated in this study.

No significant increase was noted in urea levels. However, creatinine levels at T1 and T18 increased significantly, considering the 28 animals studied, though these levels remained within the reference values. Pathological increases in urea and creatinine levels due to a decreased glomerular filtration rate (GFR) indicates kidney damage (Di Filippo et al., 2015), and creatinine is more sensitive than urea in evaluating GFR (Moreira et al., 2015). The increase shown by the animals studied was associated with exercise. As increased creatinine levels were within the reference range this increase may have stemmed from heightened phosphocreatine catabolism in muscle cells (Moreira et al., 2015).

The 28 evaluated horses did not show significant changes in plasma glucose levels; however, plasma glucose levels decreased throughout the collection period. A decrease in plasma glucose concentration was reported at the beginning of exercising tests applied to racehorses, but as exercise was applied, glucose levels increased. This behavior was explained by a slight decrease in glucose levels; as exercise continued, glucose concentrations tended to increase due to the stimulation of hepatic gluconeogenesis activation (Trilk et al., 2002). The lack of increase in plasma glucose concentrations in the present study suggests that the type of exercise practiced by the horses was not sufficient to stress-induce glucose consumption until hepatic gluconeogenesis was triggered to replenish its levels.

Triglyceride level stability indicated good conditioning in the 28 investigated animals, as this parameter could have decreased after physical effort due to using triglycerides as an energy source. Lipid reserves in horse blood can be assessed based on triglyceride and total cholesterol concentrations; however, triglycerides are the most important variable for athletic horses as they are energy sources (Melo et al., 2013). Stress or exercise leads to increased lipolysis owing to catecholamine release. Elevated triglyceride concentrations after exercising are often expected due to insulin inhibition during exercise and the hyperglycemic effect induced by circulating catecholamines and cortisol due to physical activity (Hyyppä, 2005). However, the type of exercise performed by the studied horses did not significantly increase this parameter. The level of cholesterol increase observed in this study, after exercising and resting, is dure to insulin release within 1 h after exercising. This further inhibited lipolysis when serum lipid concentrations reduced their utility as an energy source (Hyyppä, 2005).

## Conclusion

Criollo horses engaged in long-loop rodeos evaluated are desired for their high-intensity, short-duration exercises at varying speeds, their ability to carry considerable load (>25% of their body weight). The exercise intensity proved insufficient to elevate plasma glucose levels or reduce triglyceride levels for energy. Such observations, in association with significant increases in hematocrit, hemoglobin, erythrocyte, and TP levels, and with their return to baseline levels in a timely manner, have indicated that even under significant physical effort in 2 d rodeos and without controlled or standardized training regimens, horses evaluated did not undergo changes capable of indicating injuries at the cellular level; they were fit to compete in the tournament. However, it is necessary to standardize training to avoid exercise overload. Hematological and biochemical assessments can help identify horses at a higher risk of experiencing changes due to the physical effort undergone during long-loop tournaments. Furthermore, they provide information regarding the animals' responses to the effort required and can also provide information on animals' response to the required effort.

The hematological and biochemical analyzes evaluated here considered the means obtained through statistical analysis, but individuals may present changes when evaluated separately. Other studies with other animals of Criollo breed who participate in long-loop rodeo should be done to see if the findings are consistent.
